# Surmounting difficulties to provide home based neonatal care – reflections of community health workers

**DOI:** 10.1186/s12905-018-0511-6

**Published:** 2018-01-15

**Authors:** Abhijit Shrinivas Prabhughate, Pearl Tiwari, Shubhangi Sohoni, Vallaree Anant Morgaonkar, Ajay Gajanan Phatak, Somashekhar Marutirao Nimbalkar, Anagha Anand Mahajani

**Affiliations:** 1 Program Monitoring and Research, Ambuja Cement Foundation, Mumbai, 400059 India; 2 Department of Paediatrics, Pramukhswami Medical College, Karamsad, Anand-Gujarat 388325 India; 3 Central Research Services, Charutar Arogya Mandal, Karamsad, 388325 India

**Keywords:** Newborn care, Strategy, Commitment

## Abstract

**Background:**

In India, community health workers’ (CHW) effectiveness in providing home-based neonatal care (HBNC) has been well documented. The nature of challenges faced and strategies adopted while providing HBNC services need to be studied in-depth.

**Methods:**

A qualitative study to understand the challenges faced and strategies used by Sakhis (women CHW) while providing services as part of a HBNC program implemented by a non-profit organization. Data consisted of 20 in-depth interviews and three focus group discussions (FGD) with Sakhis.

**Results:**

Sakhis negotiated with the community to start working as a CHW. They faced challenges while changing behaviors at individual level and also while bringing about a change in harmful normative practices that increased chances of maternal and neonatal mortality. Managing crises at the time of deliveries and facilitating a safe delivery was the most critical challenge faced by many Sakhis.

The key strategies used by Sakhis included: proactively and persistently providing services even when they faced resistance from the woman or her family; evolving contextually suitable counseling techniques and tactics to bring about behavioral change; balancing compliance to traditional practices and promoting HBNC; defying traditional practices and assisting the woman in times of an emergency to save lives. Having on-call support from supervisors and cultivating a good working relationship with health providers facilitated effective service provision by Sakhis.

**Conclusion:**

CHWs having a strong sense of commitment can develop strategies to address challenges and provide HBNC services effectively if they also have strong supervisory support.

**Electronic supplementary material:**

The online version of this article (doi: 10.1186/s12905-018-0511-6) contains supplementary material, which is available to authorized users.

## Background

Community health workers (CHW) play a crucial role in ensuring availability of basic healthcare services. They are at the core of the public health strategies to ensure access to primary health care for all populations. The CHW are derived from the community they serve and hence are usually the point of contact for various health needs, especially emergency care. The government of India (GOI) introduced CHW called accredited social health activist (ASHA) in 2005, who have been trained to provide basic Maternal and Child Health (MCH) services during pregnancy and childbirth, and monitor the health of infants.

A landmark study by Bang et al. [1] demonstrated the effectiveness of home based neonatal care (HBNC) provided by CHW in reducing neonatal mortality in rural Indian communities. This is now widely recognized as the HBNC model and the government of India has also adopted it with the objective of reducing morbidity and mortality among women and children in the country. A distinctive feature of this model is that CHW are trained in early identification and treatment of neonatal morbidities such as asphyxia, prematurity, low birth-weight, umbilical sepsis, fever and other illnesses [2]. More than 550,000 CHW have been trained in these skills and it is expected that in the due course HBNC will be provided by all ASHA after completing their training [3]. In this context of scaling-up of HBNC in the country, we present insights from a study with CHWs who have provided HBNC as part of a decade old program implemented in rural Maharashtra. The lessons from this study will be valuable to planners and implementers involved in the scale-up.

## Literature review

Studies have examined various aspects of ASHAs’ work since their introduction in the public health system in 2005. These suggest that ASHA are primarily functioning as link workers and are accepted as service providers by the community [4]. An evaluation conducted by National Rural Health Mission (NRHM) in 16 districts of 8 states showed that ASHA have become functional in key roles related to MCH promotion – 85% of the ASHA were accompanying women for deliveries in six states; more than 70% were counseling women on all aspects of pregnancy and promotion and visiting the newborn; over 90% were also involved in promotion of institutional delivery [5].

The performance of these roles is affected by contextual factors such as existing power structure within a community, proscriptive gender norms around women’s roles, difficult geographic terrain that limits ability of the CHW to link community to healthcare services, and programmatic factors such as incentives received under different schemes [6]. Personal factors such as aptitude, communication skills, leadership and ability to reach out to the community also affect performance [7].

The processes involved in a CHW’s work in the community have received lesser attention in comparison to the focus on her performance. A qualitative study showed that outreach and health services provided by CHW are embedded in the process of developing a relationship of trust with the community [8]. It has also been observed that CHW can be effective in addressing broader social, economic and political factors influencing the health of people when they have involved in social interventions such as mobilizing women’s collectives, raising awareness on rights and engaging in social action that targeted both community and government agencies [9]. There is a lack of sufficient literature from India which details the challenges faced by CHW and the strategies adopted by CHW to overcome or mitigate these difficulties.

## Methods

### The setting

The study was conducted with CHWs called Sakhis who have worked in a MCH program initiated by Ambuja Cement Foundation (ACF) in 2005. The Sakhi program was initiated in response to the felt need of the community following participatory process involving key opinion leaders in the community. The program’s objective was to mitigate the challenge of high infant and maternal mortality in the three blocks, namely, Jiwati, Korpana and Rajura, of Chandrapur district in the state of Maharashtra, India. The structure and conduct of the program was largely based on the home based neonatal care model adopted by Bang et, al. The Sakhis were recruited from the community and underwent a series of training sessions interspersed with field practice and continuous supportive supervision provided on an as needed basis and through bi-monthly group meetings.

### Study design

**Fig. 1:**
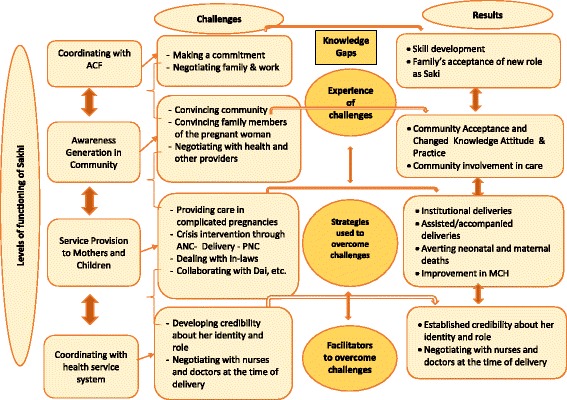
Conceptual framework

A qualitative exploratory study that was guided by the grounded theory approach was conducted. The reiterative process of conceptualization comprised of several rounds of observations and interactions with stakeholders including program staff and some Sakhis to understand the evolution of the program and the nature of their work in the community. A conceptual framework was evolved on the basis of this information, which guided further exploration in the study (Fig. 1). Key concepts in the framework included the levels of functioning of the Sakhi, challenges faced at each level, and the results achieved upon successful mitigation of those challenges. Knowledge gaps existed in regard to the Sakhi’s experience of challenges, strategies utilized by Sakhis and the facilitators of successful mitigation. These gaps were explored in data acquired from Sakhis.

### Sampling

Adopting a purposive selection strategy Sakhis with at least 2 years of experience were recruited from each of the three blocks in which the program was implemented. Overall, 35 Sakhis participated in the study [Table 1]. In-depth interviews (IDI) were conducted with 20 Sakhis and three focus group discussions (FGD) with 6–7 Sakhis in each group were conducted with 21 Sakhis. While both IDI and FGD acquired information about experience of challenges and strategies adopted by Sakhis, the FGD also focused on the Sakhis’ perception of what facilitated their effective functioning and their experience of being associated with ACF. Hence, six Sakhis who expressed interest in participating in the FGD even after completing their IDI were included in the FGD. Details of guide used for interview and discussion are available as Additional file 1.

**Table 1:** Profile of study participants

Number of study participants (Sakhis)	35
Number of Sakhis interviewed	20
Number of Sakhis participating in FGD	21^a^
Age [Mean (range)]	38 (26–55)
Years of experience of providing HBNC [Mean (range)]	6.74 (2–10)

^a^6 interviewed Sakhis also participated in FGD

### Data analysis

The interviews and FGD were conducted by three of the authors who are proficient in Marathi, the local language, and the transcripts were prepared from audio-records by a translator proficient in both languages. Data analysis was initiated after the authors had conjointly read few interviews and all FGDs and developed mutually agreed upon code definitions. Subsequently, at least two authors coded each interview and FGD using NVivo and then again conjointly identified emerging themes.

The ethics committee of the Narotam Sekhsaria Foundation approved the study.

## Results

### Creating an identity and gaining acceptance as a service provider

Many Sakhis shared that in the initial days of their work they had to be patient and work consistently for people to accept them as service providers. Some faced challenges from their own family members who perceived that a lay woman taking up the role of a paraprofessional was not consistent with the prescribed gender roles in the community. Many reported that they started working against the family’s wish, but the family gradually accepted their role after they started getting recognition from the community.

A few Sakhis shared that some communities did not accept them for various reasons such as their limited literacy and their work being unconventional for a woman. The extent to which community’s pressure could impede the functioning of Sakhis can be seen in the experience shared by a Sakhi:

*“Some people in our community were against my work as it was unusual according to them. They asked why I was working, why I was going out. They also told me to discontinue my job as I was going alone for the training for 10 days… I stopped working for some period. Then Sir (ACF supervisor) came and convinced them.”*

Another Sakhi shared that at times it took as long as 2 years for the community to start unconditionally cooperating with them. The resistance continued in the form of women not giving information about pregnancy because their families did not trust the Sakhi.

It appeared from the data that the Sakhis were able to create an identity and work in the community as a result of two main strategies that were adopted concurrently. One was of calling for community meetings involving key opinion leaders. The support to Sakhis from ACF as an institution was explained by the supervisors in these meetings, which helped to legitimize their role as a CHW. Persuading the leaders of the need for Sakhi’s work also helped in getting their endorsement for HBNC work, which in turn led to the community opening up to the Sakhi. The other strategy involved Sakhis proactively visiting families to identify pregnant women. Many families did not appreciate the Sakhi visiting and providing information about mother and child care, yet the Sakhis continued to visit them repeatedly and did not exclude them from their services, which ultimately led to acceptance by the family. The spirit of persistence can be seen in a Sakhi’s sharing:

*“Some women used to give information, but some used to say, ‘Why is she asking? What is she going to do with all this information? Is she going to give us food?’ Even after this, we didn't deter, didn’t fear.”*

### Changing community norms and cultural practices

The Sakhis shared several social norms and cultural practices followed by some communities, which increased the risk of morbidity and mortality for the mother and neonate. Some examples of peculiar practices included the community forcing the woman to conduct her delivery on her own by secluding her, keeping the new born child and the woman in separate rooms and forbidding the mother from seeing the baby for 7 days after birth, and not allowing the mother to step out of her room out of fear of being possessed by evil spirits.

A review of the data for how Sakhis facilitated a change in these practices showed that, in addition to conducting group-based educational activities that targeted change in entire community, the Sakhis meticulously conducted their intervention by treating the “family” as a unit of intervention. This appeared to be because resistance manifested most during home-visits made by Sakhis, and usually arose from many members of the family who dissuaded the pregnant woman even if she was willing to seek help from Sakhi. For instance, while explaining how family members thwarted their efforts to help the pregnant woman, a Sakhi shared that *“they (mothers-in-law) thought we gave nonsensical advice, and said that they had given birth to seven odd children but had never needed a doctor, never went to any maternity home, never took an injection”* and hence did not want the daughter-in-law to take care of health according to Sakhi’s advice. Sakhis made efforts to change views of all members including mother-in-laws, women in the family, and also actively engaged with men as they were reluctant to engage in discussions because of the gendered stereotype restricting men’s engagement in care of women during pregnancies. The strategy of focusing on family members and men was beneficial. It was evident from a Sakhi’s experience, in which people from the community disallowed her to assist a woman during delivery because they believed that the woman should be left alone at the time of delivery. In this situation, however, the husband dared to defy the community norm and allowed the Sakhi to enter the secluded room and assist his wife.

The Sakhis also encountered situations where the community threatened families and the Sakhi with sanctions such as ostracizing them or forcing them to go through cleansing practices for breaching traditional practices. The Sakhis addressed challenges emanating from such practices by relying on their knowledge and familiarity of the community’s culture. They used it to contextualize their messages and formulate explanations and counter-arguments to change myths and misconceptions associated with traditional practices. In many situations that warranted immediate action, Sakhis appeared to have judiciously balanced compliance to prevalent norms and their aim of providing services. In some situations they also risked the ire of the community by taking steps for preventing mortality, and in this process endured humiliating treatment by the community. The delicate balancing of the community’s expectations and performing their role can be seen in how a Sakhi addressed a difficult situation:

*“I used to go prior to starting of her (pregnant women’s) labour pains. After the delivery, if I wanted to touch the baby I had to burst an egg on my head and then I had to take bath. I had to return home by the route outside the village. I was not allowed to go home by the road, which passed through our village.”*

It can be seen from this sharing that the Sakhi followed the community norm in order to provide services. Overall, it was evident from the data that the grip of traditional cultural practices typically started loosening when the community witnessed a Sakhi using her HBNC tools to conduct a safe delivery, or when she saved the life of a mother or neonate in a critical situation.

### Facilitating behavior change in individuals

The Sakhis’ narration of various situations showed that they embedded counseling in routine interactions with pregnant women and families. Offering analogies that suited the social background of the women stood out as the most used method of convincing and facilitating a change in behavior. It was also evident that the Sakhis provided targeted counseling on specific issues that were relevant for a selected member of the family. For instance, a counseling interaction with a mother-in-law would be primarily focused on garnering her support for allowing the pregnant daughter-in-law to take medications. As shared by a Sakhi, in this situation the mother-in-law would be convinced by using an analogy – the need to take medication for better health would be compared with the need to change farming practice. The explanation offered was that as farmers had changed their approach to adopting modern methods of farming to get better yield, there was also a need to change the approach to giving medication for better health of pregnant woman.

A predominant misconception encountered by many Sakhis was that families believed using health care services was uneconomical because the pregnant woman would be advised to stop working in the farms. The strategy used to address this issue was of making the family re-examine the belief by providing information about potential costs and savings in seeking healthcare services. For example, to convince a family to spend on food for the pregnant woman and allow her to take rest, the Sakhi would first make the family evaluate the cost of care if the mother or child fell sick. Then she would help the family to understand how this potential cost was higher than the earnings lost because of the woman not working in the field.

The data showed that Sakhis had cleverly developed certain tactics to ensure that women followed their advice. One such tactic involved gentle confrontation and conditional provision of services. For example, a Sakhi shared that she would assertively tell the mother-in-law *“It is your responsibility to give her medicines”* in the presence of the pregnant daughter-in-law, and on the other hand tell the pregnant woman *“I will take you to the medical camp only if you take tablets”.* Another tactic was to innovatively use available material to ensure that their messages could be followed by women with low literacy. An illustration of this can be seen in the report of a Sakhi helping the woman to follow the right practice of feeding the neonate:

*“I had given her a piece of coal and told her to (make a) mark on the wall every time, when she fed the baby. She was illiterate. I told her to feed the baby for at least 10-12 times a day regularly. She used to write with the help of coal on the wall, the number of feedings. I used to see her in the evening everyday (to check if the child was fed adequately)”.*

The essential element that appeared to have led women and their family members to change their health seeking behavior was the assurance from Sakhis that they were available round-the clock to anyone in need.

### Managing crisis at the time of deliveries

Many vivid descriptions by Sakhis showed that they perceived providing assistance during deliveries as a complex event riddled with challenges posed by the social context, and not just a process involving use of their HBNC knowledge and skills. In some crises situations the Sakhi had to make judgments and decisions without much support. A challenge faced by almost all Sakhis was of arranging for a vehicle to take the woman to the hospital for delivery. The strong commitment of Sakhis and their ability to manage crisis without panicking was evident in the experiences of crisis management. For instance, in one situation the Sakhi could not get the government’s free ambulance service even after trying to get it from various nearby health facilities, and therefore paid from her own pocket to take the woman to the district hospital. Another Sakhi assisted the woman’s delivery at home and also detached the placenta applying knowledge gained from training, even though it was not her responsibility.

At times the community’s resistance and non-cooperation also posed a challenge. In one such experience shared, when a community was not willing to assist a woman in a complicated delivery and neither allowing the Sakhis to intervene, two Sakhis shared the risk in defying the community by telling them *“If you don’t want to help, don’t go but let me go and see. I will not harm your baby. It will be my responsibility, if anything goes wrong*”. When Sakhis assisted during deliveries at home, they often had to overcome their fear of being blamed by the family if the outcome of the delivery was fatal for the mother or the child. The fear experienced by a Sakhi who reached a house when a woman had delivered but not received any help is evident from the experience shared by her:

*“Her mother stayed nearby, when we tried calling her relatives, nobody came near. They said that they will not touch her. And said that you do whatever you want to do by yourself… And I said (to myself) that I will be blamed if something worst happened to that woman”.*

### Building a strong partnership with health providers

The data showed that one of the main challenges faced by Sakhis was to negotiate a space and role for themselves in the established system of both formal and informal health providers. With the *dai* (the traditional birth attendant) of the community, as facilitated by the ACF supervisors, the approach adopted was a clear de-lineation of the roles. When the *dai* was present for deliveries, the Sakhis focused primarily on providing post-partum services and using specific HBNC techniques needed for averting mortality. With *dai* and Anaganwadi workers, the Sakhis attempted to bring about a change in practices by taking a supportive stance. For example, in a village, Sakhis coaxed Anganwadi workers to identify and report cases of severely and moderately malnourished children and also assured them of support in providing care to the children.

The overall approach to working with formal providers appeared to be focused on developing a cordial working relationship. A Sakhi shared that doctors did not trust them initially and questioned their presence at the time of delivery, but accepted them when they witnessed the Sakhi using her skills. In this process, possessing the HBNC kit appeared to have helped the Sakhis in partnering with the doctors. This was because some doctors were not aware of the HBNC and hence didn’t know how to use it, and in case of some, equipment was not available or was dysfunctional in their facility. Some Sakhis also shared experiences in which they had to insist with a resistant doctor or nurse to be allowed to use HBNC tools for resuscitating the child after the doctor had given up hope of revival. However, as reported by the Sakhis, such encounters usually resulted in getting the provider’s appreciation, and helped them to develop a strong working relationship. The partnership in the relationship with the doctors was reflected in many experiences which showed that doctors had started allowing Sakhis inside the labor room at the time of delivery, some had shared their personal contact number and made themselves available to Sakhis at any time, others gave referral notes to mothers to seek follow-up care from Sakhis in the village, and at times some even went beyond their mandate to support the Sakhi in managing crises, which contributed to saving many lives of mothers and newborn.

### Getting support from supervisors

Many Sakhis opined that the quality and approach of their training had helped them build their skills. They asserted that they were able to use the HBNC kit because it was made available during the training, in which they were oriented to the kit and then asked to practice for a few weeks along with their supervisors, and then trained again in refresher training sessions. Along with the training, Sakhis found on-the-job support provided by their supervisors in the initial period as most valuable and attributed their skill development and confidence to this supervision. Another source of support for many Sakhis was the bi-monthly meetings conducted by their supervisors. How these meetings served the purpose of getting information and clarifying doubts can be seen in a Sakhi’s description of a typical meeting:

*“We used to report each and everything. I used to tell Bhau (supervisor) about everything. And if I didn’t understand anything, I used to ask information regarding the same. We used to get information from Sakhi in other villages and vice versa. If something we did was wrong, we used to correct it by knowing from Sakhis in other villages”.*

Most Sakhis also considered availability of any-time support from the supervisor as vital to their effective functioning in the community. They requested for such support by calling the supervisors and asking them to be present with them in difficult situations or sought guidance for specific problems, which, as Sakhis emphasized in a FGD, was always obliged to by the supervisors. Other experiences also reflected that association of supervisor and Sakhi was not limited to getting technical information and guidance. In many difficult situations such as when the family compelled the Sakhi to discontinue her work or doubted her integrity because she would be away for long duration of time for her work, the supervisors visited their homes and vouched for the genuineness of Sakhi’s work. Overall, the narratives revealed that Sakhis had developed a bond with their supervisors which helped them to function effectively, regardless of the difficulties they faced.

## Discussion

The CHW are the fulcrum of the HBNC model that is in the process of being scaled up across the country. We conducted an in-depth inquiry to understand the challenges faced by CHW and their mitigation strategies in providing HBNC. Even though the experiences shared by Sakhis in our study were in a particular geographic and temporal context, several insights that can guide the scale-up process were acquired.

As reported in earlier studies, [6, 8] our study showed that Sakhis faced several challenges due to gender proscriptions, power dynamics in the community, difficulties due to difficult terrain and lack of availability of facilities such as proper transportation required at the time of delivery. It also showed that community norms and traditional practices can be a potentially strong barrier for uptake of services offered by the CHW. Most importantly, the study showed that some of these challenges could actually translate into a community’s non-acceptance of the CHW. It highlighted that community’s acceptance may become a pre-requisite for effective provisioning of HBNC services in the community. This underscores the importance of CHWs developing a relationship of trust with the entire community and not limiting the scope to working with the woman or child as a beneficiary of her services.

The Sakhis had ingeniously developed messages and used several techniques such as developing analogies for bringing about a change in health behaviors. They were able to make their messaging compatible to the socio-cultural background of the people and present it in a manner acceptable to the communities. Using these techniques and making their services available round-the-clock, the Sakhis reached out to the most marginalized and hard to reach communities. This illustrates how CHW can use their competence drawn from their aptitude and experience of working with communities to adapt the knowledge they have received as part of their training. In other words, the CHW can bring in valuable additions from their personal competence, which otherwise may be seen as a limiting factor by program planners.

The narratives about deliveries reaffirmed that CHWs can effectively use HBNC skills to avert neonatal mortality. They often actively facilitated safe deliveries even in crises situations. It is noteworthy that the crises were usually due to social and contextual factors that created obstacles for the Sakhi and not due to a lack of knowledge and skills of Sakhis. With strong commitment, confidence and support from supervisors, they were able to surmount several difficulties, guide families, and make critical decisions on their behalf. It is most likely that they could overcome their fear of being blamed and use HBNC tools and techniques because they had established their credibility through a long process of engagement with community and other providers. It also reflects that it is not adequate for CHW to possess only HBNC skills; they need to be prepared for crises situations and have a repertoire of different skills to mitigate these situations.

An important insight from this study is also that a good working relationship between the formal health system and CHW can be developed to meet the objective of averting maternal and neonatal mortality. In case of Sakhis it appeared to have grown over a period of time as doctors started understanding and recognizing the potential of Sakhis. This shows that in Indian context where a significant gap exists between the formal health-care system and the community, nurturing partnerships between CHW and formal health care providers in their day-to-day work can go a long way in closing this gap.

A key learning from this study is that CHW can nurture a relationship with the community if they receive on-going support in not only developing HBNC knowledge and skills, but also in addressing challenges they face in delivering HBNC services. Such support can be provided by creating platforms such as bi-monthly meetings where CHW get opportunity to validate their experiences and learn from their peers. In other words, consistent and committed high-quality supervision can be a key determinant of CHW’s performance.

## Conclusion

The Sakhi experience shows that their genuine interest in improving the lot of the women in their community led them to develop solutions and negotiate with community members and family to achieve the desired outcomes. In the process their belief in themselves grew and of the health ecosystem in them also grew. The role of a supportive network would be crucial as they rely mainly on the supervisors when faced with operational and clinical difficulties. While we show the effectiveness of the Sakhi program, it may not be extended to similar instances of ASHAs. But we do show that if there is no improvement in areas where ASHAs work, it would be of interest for the system to conduct a similar inquiry to tease out problems that can be solved. Our model can thus be used as a comparison to other areas that utilize CHWs in similar capacities. Our current work reiterates the belief of public health managers in CHWs as the last mile connectivity for maternal and child health.

## Additional file

Additional file 1: Guide for interview and discussion with Sakhis. (DOCX 14 kb)

## Abbreviations

ACF: Ambuja Cement Foundation
ASHA: Accredited social health activist
CHW: Community health workers
CSR: Corporate social responsibility
FGD: Focused group discussion
GOI: Government of India
HBNC: Home based neonatal care
MCH: Maternal and child health
NGO: Non-Governmental Organization
NRHM: National Rural Health Mission
