# Supplementary material for: Surmounting difficulties to provide home based neonatal care – reflections of community health workers
Source: BMC Womens Health. 2018 Jan 15;18:17. doi: 10.1186/s12905-018-0511-6 (PMC5769228; doi:10.1186/s12905-018-0511-6)
Supplement: Additional file 1: — Guide for interview and discussion with Sakhis. (DOCX 14 kb) [file 12905_2018_511_MOESM1_ESM.docx]

Guide for Interview and Discussion with Sakhis

Instructions for Interviewer/Facilitator

The interview / FGD should be initiated after giving your introduction and seeking consent from the interviewee/members. The actual interview/FGD should start after ensuring that the interviewee/respondents have become comfortable to share their experiences.

During the interview/FGD the broad questions should be asked and the specific areas should be explored only if the information shared by the individual Sakhi/group in the natural course of discussion is too sparse.

***Broad Question and Probes***

1. How did your work on HBNC start?
   - Who communicated and how did you get involved?
   - What made you feel like joining HBNC?

*Specific areas of exploration:*

- Enrollment as Sakhi
  - What were you doing before joining ACF?
  - What motivated you to become a Sakhi?
  - How was the support from your family for you to become Sakhi?
  - How did you manage family when you started trainings for Sakhi?

***Broad Question and Probes***

1. How was your experience once you started working as a Sakhi?
   - Can you tell us anything that happened while working as a Sakhi that has remained in your memory?
   - How did you manage these situations? What actions worked and what did not work?
   - Experience at different levels – individual, community, mothers and family, health providers

*Specific Areas of Exploration*

- Enrollment as Sakhi
  - What were you doing before joining ACF?
  - What motivated you to become a Sakhi?
- Experience of capacity building
  - What were the things you were taught?
    - How were they taught?
  - How did you find the training? What training components did you find beneficial?
  - Do you feel that the training made any difference to you?
    - What? How?
- Experience of working with communities
  - Experience of introducing HBNC in community
    - How did you initiate work in the communities you were assigned? What was your experience? How did you deal with the challenges?
  - What did you have to do to increase early birth registration and health check-ups in the community?
- Experience of working with mothers and their families
  - How did you make sure that identified pregnant women got your messages? How did you motivate them to seek services?
    - Role of different family members
    - Preparing family members and other key stakeholders (e.g. dai) to inform at the time of delivery
    - Role of dai or other informal health providers
    - Tactic used for dealing with difficult situations and people
  - Delivery
    - Delivery at home and institution – difference in experiences
    - Has there been any difference in the villages with regard to pregnancy and delivery after you started working?
    - Experience of working with doctors and other health staff in PHC
    - High risk cases assisted, experience of family and other stakeholders (quacks, dai, doctors)
  - Post-natal care
    - Visits and issues addressed – hypothermia, breastfeeding problems, diarrhea, pneumonia, pus formation in navel, sepsis
    - Response of mothers and other family members

**TO BE EXLPORED ONLY IN FGD**

***Broad Question and Probes***

1. What were the things (factors) that helped you work effectively?
   - Experience with ACF
   - Supervision
   - Other factors
2. What changes have come about since you started work as a Sakhi?
   - Changes in your working environment
   - Changes in yourself
